# Optically controlled coalescence and splitting of femtoliter/picoliter droplets for microreactors†

**DOI:** 10.1039/d2ra02230c

**Published:** 2022-06-22

**Authors:** Mingcong Wen, Benjun Yao, Shun Yuan, Weina Zhang, Yao Zhang, Guowei Yang, Hongxiang Lei

**Affiliations:** School of Materials Science and Engineering, Nanotechnology Research Center, State Key Laboratory of Optoelectronic Materials and Technologies, Sun Yat-sen University Guangzhou 510275 China leihx@mail.sysu.edu.cn; School of Information Engineering, Guangdong University of Technology, Guangdong Provincial Key Laboratory of Photonics Information Technology Guangzhou 510006 China; Institute of Nanophotonics, Jinan University Guangzhou 511443 China zhyao5@jnu.edu.cn

## Abstract

Microreactor technology has attracted tremendous interest due to its features of a large specific surface area, low consumption of reagents and energy, and flexible control of the reaction process. As most of the current microreactors have volumes of microliters or even larger, effective methods to reduce the microreactors' sizes and improve their flexibility and controllability have become highly demanded. Here we propose an optical method of coalescence and splitting of femto-/pico-liter droplets for application in microreactors. Firstly, two different schemes are adopted to stably trap and directionally transport the microdroplets (oil and water) by a scanning optical tweezing system. Then, optically controlled coalescence and splitting of the microdroplets are achieved on this basis, and the mechanism and conditions are explored. Finally, the microdroplets are used as microreactors to conduct the microreactions. Such microreactors combine the advantages of miniaturization and the multi-functions of microdroplets, as well as the precision, flexibility, and non-invasiveness of optical tweezers, holding great potential for applications in materials synthesis and biosensing.

## Abstract 2

Optical trapping, transportation, coalescence and splitting of femto-/pico-liter microdroplets are realized based on a scanning optical tweezing system. On this basis, the microdroplets are used as microreactors to conduct the microreactions.

## Graphical abstract

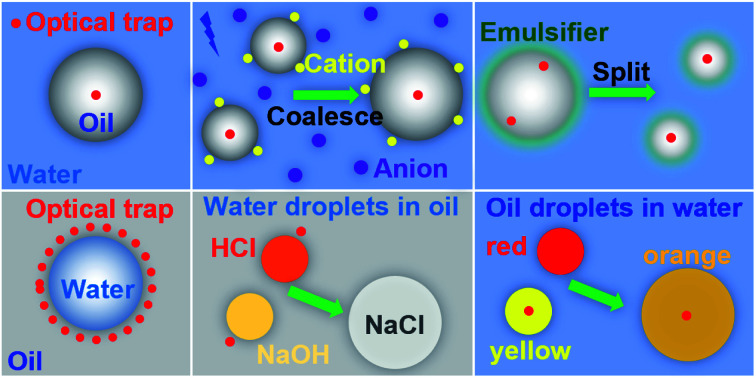

## 1. Introduction

Due to their unique advantages in diverse aspects, microreactors have attracted great research interest.^1–5^ Firstly, they have very large specific surface areas, which improves the efficiency of mass transfer and heat exchange, thereby increasing the rate and yield. Secondly, their small volume can reduce the consumption of reagents and energy. In addition, they can control the reaction process more precisely, so that dangerous or highly exothermic reactions can also be handled more safely. The features above mean the microreactors have great prospects in chemical and biological analysis.^6–8^ So far, microreactors have been developed in microchannels, liquid marbles and microdroplets. In the microchannel reactors,^9–12^ many tiny channels were precisely fabricated on a solid substrate for the flow and reaction of liquids. Such a microreactor has excellent mass transfer and heat exchange capacity, but it possesses high fabrication cost, low control accuracy, and high risk in cross contamination or quality loss. Moreover, when the reaction involves solid particles or precipitates, it is easy to cause a channel blockage. Recently, other novel microchannels-based microreactors combining the advantages of microplasmas and microfluidics have been reported,^13,14^ which can effectively avoid unwanted particle aggregation and reduce the source of contamination. The liquid marble reactor was formed by encapsulating a liquid with hydrophobic particles,^15–17^ which avoids the direct contact between the liquid and the external environment as well as the cross-contamination. However, such microreactors require complicated operations to open the liquid marbles for reaction, such as applying a magnetic field or acoustic levitation.^18–20^ The microdroplet-based reactors in oil or water droplets dispersed in liquid or air are a sort of very simple microreactors.^21–24^ The controlled initiation was based on the stable manipulation of droplets, including its trapping, transporting, coalescence and splitting, which could be achieved by external fields such as magnetic^23^ and ultrasonic waves.^24^ This method can also avoid the contact between the droplets and the solid surface, which is more flexible and controllable to microreaction and the related analysis. However, most of microdroplets-based reactors had volumes of microliters or even larger. For microdroplets with femtoliter/picoliter volumes, more precise manipulation methods are required.

Optical tweezers, which use a highly focused laser beam to trap and manipulate microobjects through photon momentum transfer,^25^ has been used to trap and manipulate the femtoliter/picoliter microdroplets in air^26^ or deform and split micron-sized emulsion oil droplets in water.^27^ Recently, Chen^28^*et al.* reported the stable trapping of the intracellular lipid droplets based on optical tweezers, which were applied as microlenses to strengthen microscopic imaging and detect the intra- and extracellular signals. Compared with other methods, microdroplet manipulation based on optical tweezers has huge advantages, such as non-contact remote manipulation, non-invasiveness, high precision, high flexibility and high biocompatibility. Even so, the optical tweezers become very much noneffective to coalesce oil droplets dispersed in water and manipulate water droplets dispersed in oil. The former is due to a strong electrostatic repulsive force between the oil droplets induced by the orderly arrangement of hydrophilic and hydrophobic groups on the surface of oil droplets, which is not enough to be overcome by the optical force. The latter is because of a lower refractive indices of water droplets than that of the surrounding. Lorenz^29^*et al.* used optical tweezers to trap and coalesce water droplets in oil by adding other solute into the water microdroplets to increase its refractive index, but the method may cause side-effects and affect quantitative microreaction analysis. As far as we know, there have been few reports of effective coalescence of femtoliter/picoliter droplets with arbitrary refractive indices and their application in microreactors.

Therefore, in this work, we proposed a novel universal and multifunctional method of coalescence and splitting of femtoliter/picoliter droplets for further applications to microreactors. Firstly, with the assistance of a scanning optical tweezers system, two different schemes were adopted to realize the stable trapping and directional transportation of oil microdroplets and water microdroplets, respectively. On this basis, the coalescence and splitting of microdroplets were realized, which played a vital role in the controlled initiation of the microreactions. And the corresponding mechanism and realization conditions of the coalescence and splitting were also explored. Finally, the microdroplets were used as microreactors to conduct microreaction analysis. In the whole experimental process, no additional solute was introduced into the microdroplets, which ensures the purity of the droplet solute. These optically controlled microreactors based on femtoliter/picoliter droplets combined the advantages of miniaturization, flexibility, functionality of the microdroplets, as well as the accuracy, flexibility, and non-contact and non-invasiveness of optical tweezers, which has huge application potentials in material synthesis and biosensing.

## 2. Experimental design

**Fig. 1:**
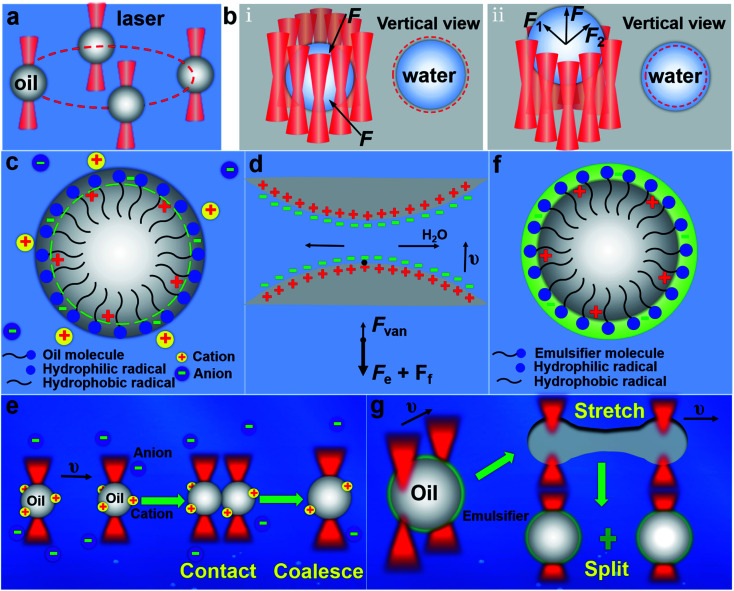
Mechanisms and experimental design. (a) The trapping and transportation of oil droplet. (b) The trapping and transportation of water droplet. (c) The surface of oil droplets in ionic solution. (d) The force between the approaching oil droplets. (e) The coalescence process of oil droplets. (f) The surface of oil droplets in the emulsifier solution. (g) The splitting process of oil droplets. The red dotted line represents the optical trap trajectory, and the red dumbbell represents the Gaussian beam.

Fig. 1 shows the schematics of the experimental principle. All the experiments in this work were performed under the scanning optical tweezers system (see Fig. S1 in ESI† for details). Microdroplets with a volume ranging from femtoliters to picoliters, including oil droplets and water droplets, were formed by mixing oil and water under the action of ultrasound. For the oil droplet with a refractive index greater than that of the surrounding, the optical force acting on it by a highly focused laser beam from the optical tweezers system is an attractive force (see Fig. S2a in ESI† for details), which makes the oil droplet be directly trapped and then transported in a directional manner, as shown in Fig. 1a. For the water droplet with a lower refractive index than that of the surrounding, the optical force acting on it is shown as a repulsive force (Fig. S2b in ESI† for details), so the same method cannot be used to trap the water droplets. To achieve the stable trapping of the water droplets, a quasi-static optical trap with a circular trajectory is constructed and then placed on the periphery of the water droplet. In this case, the surrounding repulsive force can confine the water droplet within the circular trajectory, and thus the water droplet can be trapped stably, as shown in Fig. 1b(i). And the water droplet can be further transported in the horizontal plane (*x*–*y* plane) by moving the quasi-static optical trap accordingly. Reducing the diameter of the circular trajectory (slightly smaller than that of water droplet) and adjusting the relative position of the laser focal plane and the image plane (Fig. 1b(ii)), the water droplet can be pushed and transported along the longitudinal direction (*z*-direction). Thus, the three-dimensional (3D) trapping and transportation of water droplets with a lower refractive index than that of the environment can be achieved. The stable trapping and controlled transportation of microdroplets make it possible for the coalescence and splitting of droplets.

Due to the different properties of oil and water microdroplets, there are obvious differences in their coalescence and splitting. For the oil droplets dispersed in the water, it's more complicated and thus it needs special processing to implement the coalescence and splitting. Under the shearing action of ultrasound and liquid phase, the hydrophilic groups of their surface molecules are arranged outwards while the hydrophobic groups inward, as shown in Fig. 1c. This directional arrangement makes the positive and negative charge centers of the surface molecules deviate and thus the oil droplet surface has a certain charge, which causes an electrostatic repulsive force between the oil droplets to hinder the coalescence of oil droplets. The oil droplets will be coalesced only when the electrostatic repulsive force is smaller than the optical force of the optical tweezers. To make the coalescence of the oil microdroplets easier, two methods were proposed to reduce the electrostatic repulsive force between the oil droplets. One is by adsorbing the opposite charge in the ionic solution and another is by reducing the orderly arrangement of hydrophilic and hydrophobic groups on the surface of oil droplets, such as exciting the fluorescent group of the oil droplets. When two oil droplets approach each other at a certain speed, apart from the optical force, the oil droplets are subjected to the action of three forces:^30–32^ the viscous resistance *F*_f_ exerted by the liquid phase, the electrostatic repulsive force *F*_e_ caused by the same electrical properties on the surfaces of the two oil droplets, and the intermolecular force *F*_van_, as shown in Fig. 1d. According to the dominant force, the coalescence process of oil droplets can be divided into the following three steps (Fig. 1e).^30,33,34^ Firstly, the two oil droplets approach each other at a certain speed under the action of the optical force. This step is mainly subject to viscous resistance *F*_f_. Secondly, a liquid film is formed between the two droplets, which is continuously reduced to a critical thickness under the squeezing action of optical force. The dominant force of this step is the electrostatic repulsive force *F*_e_ between the droplets, which is much larger than viscous resistance *F*_f_. Thirdly, the liquid film is broken and the oil droplets are coalesced under the actions of optical force and *F*_van_. The above is the coalescence mechanism and realization process of oil microdroplets dispersed in water. Similarly, their splitting process also needs special processing. Due to the presence of interfacial tension, the surface oil molecules tend to shrink toward the interior of the oil droplets. The optical force exerted on the oil droplets is far from enough to overcome the interfacial tension of the oil droplets. But when there exists a suitable emulsifier in the solution, a layer of emulsified film will be formed on the oil droplet surface to replace the water phase to enclose the oil droplets, as shown in Fig. 1f. Due to the existence of the emulsified layer, the interfacial tension of the oil droplets is reduced.^27^ When it decreases to a certain value, the oil droplet can be stretched, deformed and split into two daughter oil droplets under the action of optical force, as shown in Fig. 1g. For the water droplets dispersed in the oil, the electrostatic repulsive force between water droplets is small enough to be overcome by the optical force, which is mainly because the water molecule is short and the surface molecules of water droplets do not have obvious directional arrangements like the oil droplets. Therefore, under the pushing of optical force, the two water droplets can directly approach each other and then they are coalesced after finishing the liquid film drainage.

## 3. Results and discussions

### 3.1 Trapping and transportation of microdroplets

**Fig. 2:**
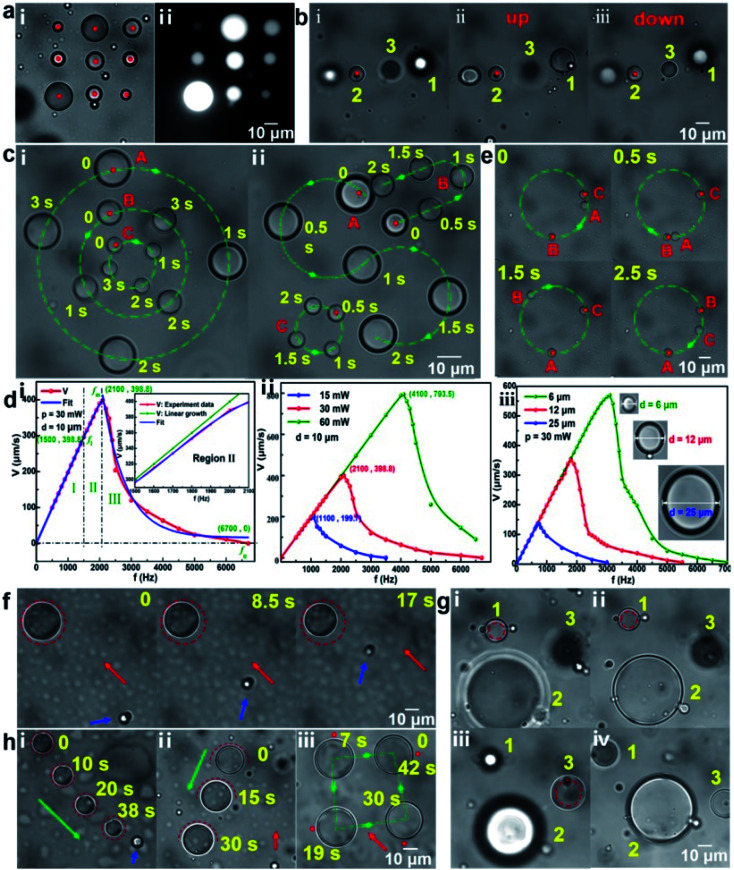
Trapping and transportation of microdroplets. (a) Optical micrograph (i) and the corresponding fluorescence image (ii) of nine trapped crude oil droplets in water medium. (b) Transportation of silicone oil droplets along the *z*-direction. (c) Various directional transportation of silicone oil droplets on *x*–*y* plane. (d) Transportation speed of oil droplets with the different laser scanning frequency (i), laser power (ii), and size of oil droplet (iii). (e) Transportation of silicone oil droplets in sequence on a circular trajectory. (f) Trapping of water droplets in the flow. (g) Transportation of water droplets along the *z*-direction. (h) Directional transportation of water droplets on the *x*–*y* plane. The red dot represents an optical trap, the green and red dashed lines represent the trajectory of optical trap, the green arrow represents the transportation direction, the red arrow represents the flow direction of the ambient fluid and the blue arrow represents a reference object.

Microdroplets with a volume ranging from femtoliters to picoliters (the corresponding diameter ranging from several to tens of microns), including oil droplets and water droplets, were formed by mixing oil and water under the action of ultrasonic waves. Oil droplets dispersed in the water could be directly trapped by a strongly focused Gaussian beam. Taking crude oil droplets in water medium as example, as shown in Fig. 2a(i), nine optical traps were simultaneously set to stably trap and arrange nine crude oil droplets in a nine-square grid pattern. Here, the red dot represents the position of optical trap and the laser power of each optical trap is about 20 mW. Fig. 2a(ii) shows the corresponding fluorescence image of the trapped crude oil droplets, which can further prove that a stable oil droplet dispersion solution could be prepared using a simple ultrasonic method. Taking silicone oil droplets in water medium as another example, the stable trapping and 3D directional transportation could be also realized using the scanning optical tweezers system. Through adjusting the focal plane of the laser beam up and down, the trapped silicone oil droplet 2 in Fig. 2b(i) could be transported along the *z*-direction to the planes where the silicone oil droplet 1 (above the droplet 2, Fig. 2b(ii)) and silicone droplet 3 (below the droplet 2, Fig. 2b(iii)) were located, respectively. Fig. 2c shows the various directional transportation of silicone oil droplets on the *x*–*y* plane. By setting the dynamic circular optical traps with different scanning directions, silicone oil droplets could be transported in different directions simultaneously with a same speed of 0.28 r s^−1^ (Fig. 2c(i)), in which oil droplets A and C were rotated clockwise while droplet B was rotated counterclockwise. As another example, silicone oil droplets could be also simultaneously transported along different trajectories (round, S-shaped, U-shaped) (Fig. 2c(ii)). The transportation speed of the oil droplets mainly depends on the laser scanning frequency, the laser power and the droplet size, as shown in Fig. 2d. For the same size of oil droplet and the same laser power, the change of the transportation speed with the scanning frequency can be divided into three regions (Fig. 2d(i)). In region I, the transportation speed is proportional to the scanning frequency with a linear growth range of 0–*f*_l_. So, it is generally selected as our working region to make sure a controlled transportation of oil microdroplets. In region II (the inset of Fig. 2d(i)), with the increasing scanning frequency, the transportation speed deviates from the linear growth until it reaches a maximum speed (the scanning frequency is *f*_m_), which is caused by the increasing resistance of the droplets with the increasing transportation speed. In region III, as the scanning frequency continues to increase, the time acting on the droplet for the laser becomes shorter and the entire acceleration process of the droplet cannot be completed, so the average transportation speed of droplet begins to decrease. When the scanning frequency is greater than a specific frequency *f*_0_, the transportation speed decreases to 0. In this case, the laser scanning frequency is high enough and the scanning interval of the same point is too short, so the optical traps can be regarded as a quasi-continuous (quasi-static) optical trap, *i.e.* there exists a laser beam on each trajectory point all the time and the laser power is equally divided. Note that *f*_l_, *f*_m_ and *f*_0_ could be increased by increasing laser power (Fig. 2d(ii)) and decreasing the size of microdroplet (Fig. 2d(iii)). In addition, oil droplets could be transported one by one on the same trajectory. As shown in Fig. 2e, oil droplets A, B and C are transported in sequence on a circular trajectory. In this way, when an oil droplet reaches the designated area, it can pass the task onto the next oil droplet, preventing accumulation and blockage. The detailed trapping and transportation process of oil droplets corresponding to Fig. 2c and e are shown in ESI Video (Video 1).†

As mentioned above, when the laser scanning frequency is high enough (lager than *f*_0_), a quasi-continuous optical trap with a circular trajectory can be constructed around the water droplets. It means that there exist a series of focused Gaussian beams on the trajectory points at the same time, and these beams produce an optical potential well within the circular trajectory, which can be used to trap and transport water droplets in oil medium. Taking water microdroplets in the silicone oil medium as an example, Fig. 2f–h shows the stable trapping and 3D transportation. When the circular optical trap was set on the periphery of the water droplet, the water droplet was trapped stably, as shown in Fig. 2f. To verify the trapping stability, a flow with a rate of ∼3 μm s^−1^ was introduced. Here, the red arrow presents the direction of the flow and the blue arrow points to the flowing particle as a reference marker. Obviously, the trapped water droplet maintained a good stability within 17 s. It should be pointed out that, 3 μm s^−1^ is not the limit of the background flow rate. At the constant viscosity of fluid environment in this work, the limit is mainly related to the laser power and the size of the water droplets (see Fig. S3 in the ESI† for the discussions). The laser power was set as 80 mW here, which was equally divided onto the trajectory points. Reducing the circular trajectory diameter slightly (smaller than that of water droplet) and adjusting the relative position of the laser focal plane and the image plane, water droplets could be transported directionally along the *z*-direction, as shown in Fig. 2g(i–iv). Water droplet 1 (below droplet 2) and droplet 3 (above droplet 2) were transported to the plane that droplet 2 is located, respectively, which is consistent with the principle mentioned in Fig. 1b(ii). In addition, the directional transportation of water droplet on the *x*–*y* plane can be also realized. Fig. 2h(i) shows a trapped water droplet within the circular optical trap could be transported along the direction of green arrow. The blue arrow points to the microparticle stuck to the substrate as a reference marker. The transportation speed was calculated as ∼2.0 μm s^−1^. In addition to the circular optical trap, other trajectory optical traps, such as semicircular and point, could be also used to transport water droplets, as shown in Fig. 2h(ii) and (iii), respectively. The corresponding speed was calculated as 2.3 and 4.2 μm s^−1^, respectively. Since the water droplets are subjected to opposite repulsive forces on both sides during the process of transportation, the transportation speed is slow using the circular optical trap. The point optical trap has a relatively faster pushing speed but the direction manipulation is unstable. In contrast, the semicircular optical trap can be used to transport water droplets more comprehensively. The detailed trapping and transportation process of water droplets corresponding to Fig. 2f and h are also shown in ESI Video (Video 2).† Additionally, it should be pointed out that, a large laser power used in the experiment is easy to cause a photothermal effect. For example, a slight shrinkage of the water droplet occurred in Fig. 2f, which was caused by the photothermal effect. Due to the introduction of the flow, the position of the trapped water droplet deviated from the center of the circular optical trap to the edge of the trap. In the case, the laser beam with the optical power of 80 mW would partially act on the water droplets. Because of a relatively strong light absorption of water at the 1064 nm, the photothermal effect and then the slight shrinkage of the water droplet occurred. Further experimental results show that when the laser power was less than 100 mW, the mass loss of the water droplets due to the photothermal effect could be negligible in a stationary liquid environment in this work, especially for semicircular or point optical trap. Additionally, it is also possible to control more complicated droplets, like W/O/W, O/W/O (see Fig. S4 in ESI† for the details).

### 3.2 Coalescence and splitting of microdroplets

**Fig. 3:**
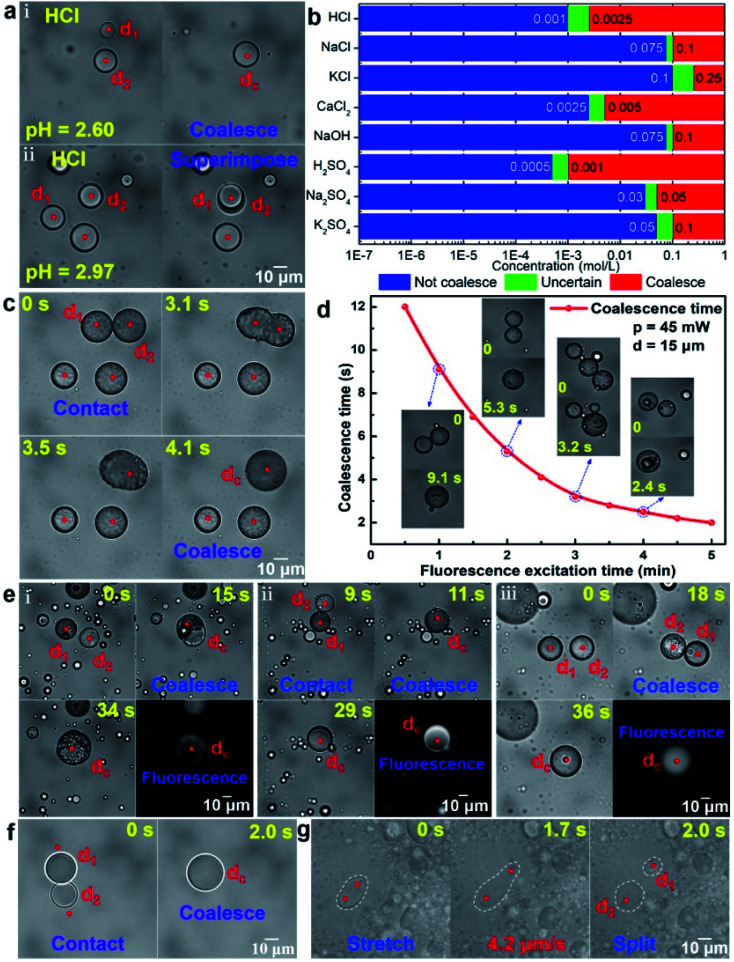
Coalescence and splitting of microdroplets. (a) The coalescence of silicone oil droplets in HCl solution. (b) The coalescence of silicone oil droplets in different ionic solutions. (c) The coalescence of crude oil droplets under fluorescence excitation. (d) The coalescence time of crude oil droplets as a function of the fluorescence excitation time. (e) The coalescence of two different kinds of oil droplets. (i) The coalescence of crude oil and silicone oil droplets under fluorescence excitation. (ii) The coalescence of crude oil and silicone oil droplets in NaCl solution. (iii) The coalescence of crude oil and soybean oil droplets under fluorescence excitation. (f) The coalescence of water droplets. (g) The splitting of oil droplets in the emulsifier solution. Each red dot represents an optical trap, and the white dashed lines represent the outline of the oil droplets.

Microdroplets have great application potential in the fields of biological analysis, micro/nanoparticle synthesis and functional material preparation.^23^ The coalescence of droplets is the foundation of these applications. However, since the optical force supplied by the optical tweezers is insufficient to overcome the electrostatic repulsive force *F*_e_ between the oil droplets, the oil droplets in deionized water cannot be coalesced. When the oil droplets are dispersed in the ionic solution, some oppositely charged ions will be adsorbed on the surface of the oil droplets, which can reduce the electrostatic repulsive force *F*_e_. The coalescence of oil droplets occurs when the ion reaches a certain concentration at which the electrostatic repulsive force *F*_e_ and the viscous resistance *F*_f_ can be overcome by the optical force. Otherwise, the coalescence will not occur. Taking the silicone oil droplets in HCl solution with the different ionic concentrations as an example, Fig. 3a shows the completely different results. Under the action of the optical force, the silicone oil droplets d_1_ and d_2_ can be coalesced as a new oil droplet d_*c*_ in the HCl solution with a pH of 2.6 (Fig. 3a(i)). On the contrary, the silicone oil droplets in the HCl solution with a pH of 2.97 cannot be coalesced but superimposed. In order to further investigate the relationship between the ion concentration and the coalescence of silicone oil droplets, more experiments have been performed (see Fig. S5 in ESI†), and the range of ion concentration suitable for the coalescence of silicone oil droplets in different ion solution is shown in Fig. 3b. In HCl, NaCl and KCl solutions, it is found that the concentration required for the coalescence of oil droplets is the smallest in HCl solution and the largest in KCl solution. The solute concentrations required for the coalescence of oil droplets in H_2_SO_4_, Na_2_SO_4_ and K_2_SO_4_ solutions are about half of those in HCl, NaCl and KCl solutions, respectively. These results show that the cation in the solutions could reduce the electrostatic repulsive force *F*_e_ between oil droplets. Moreover, the order of the ability to reduce *F*_e_ is H^+^ > Na^+^ > K^+^, which is mainly because for the same concentration and number of charges of the ions, the smaller the ion radius, the closer the cation is to the surface of the oil droplet and the more *F*_e_ is offset. The coalescence of silicone oil droplets in NaCl solution and NaOH solution is similar, which also indicates that the cations played a key role in the coalescence process. The coalescence of silicone oil droplets in CaCl_2_ solution requires a much lower cation concentration than that in KCl solution, which is because for the same ion radius and concentration, the more charges the ions carry, the more *F*_e_ is offset and the easier the coalescence is.

In addition to adsorbing the opposite charge in the ionic solution, reducing the orderly arrangement of the hydrophilic and hydrophobic groups of the surface oil molecules can also reduce the electrostatic repulsive force *F*_e_ between oil droplets. Some oil droplets with fluorophores, such as crude oil, can easily be coalesced under the action of optical force after fluorescence excitation, as shown in Fig. 3c. Before the operation of coalescence, the trapped crude oil droplets were irradiated with time of about 150 s by the blue light, which could make the smooth surface of the oil droplets become rough. Thus, the arrangement regularity of the surface oil molecule and then the electrostatic repulsive force *F*_e_ between oil droplets were reduced. In this case, when moving the trapped crude oil droplets to contact with each other by the optical force, the two oil droplets (taking the droplet d_1_ and d_2_ as example) could be coalesced within 4.1 s. Further experiments indicated that the coalescence time of crude oil droplets was closely related to the fluorescence excitation time. Taking two crude oil droplets with the similar diameter (*d* = 15 μm) as an example, Fig. 3d shows the corresponding relationship between the fluorescence excitation time and their coalescence time at the optical power of the trapping laser *p* = 45 mW. The longer the fluorescence excitation time, the worse the orderly arrangement of the hydrophilic and hydrophobic groups of the surface oil molecules, the smaller the electrostatic repulsive force *F*_e_ between oil droplets and the faster the coalescence of oil droplets. Based on the above working mechanism and method, two different kinds of oil droplets can also be coalesced by the optical force in ion solution or under fluorescence excitation, as shown in Fig. 3e. Fig. 3e(i) and (ii) show the optically controlled coalescence process of two immiscible oil droplets (crude oil and silicone oil as examples) under fluorescence excitation and in NaCl solution, respectively. Both of them formed an oil-in-oil composite oil droplet finally. This is mainly because the Marangoni effect^35–37^ caused by the difference in interfacial tension makes the crude oil droplet with a lower interfacial tension move along the surface of the silicone oil droplet with a higher interfacial tension until the silicone oil droplet is completely covered. For two mutually soluble oil droplets, such as crude oil and soybean oil, the existence of the Marragoni effect could promote the diffusion of oil molecules, and a uniform oil droplet was finally formed after their coalescence (Fig. 3e(iii)). The detailed coalescence process of oil droplets corresponding to Fig. 3a, c and e are also shown in ESI Video (Video 3).† For the water droplets dispersed in the oil, the electrostatic repulsive force between them was small and easily overcome by the optical force, so two water droplets could be directly contacted and then coalesced under the pushing of optical force. Fig. 3f shows two small water droplets dispersed in the silicone oil were directly coalesced into a large water droplet under the pushing of the optical force from the optical tweezers (see ESI Video (Video 4)† for detailed coalescence process of water droplets), which is consistent with the above theoretical analysis.

In addition to the coalescence, the splitting of oil microdroplets could be also achieved. Adding a suitable emulsifier to the oil droplets solution can make the rigid oil droplets flexible, thus the oil droplets can be stretched and deformed under the pulling of optical force.^27^ Taking toluene oil droplets dispersed in water containing OP-10 emulsifier as an example, one end of a toluene oil droplet was fixed by an optical trap, and the other end was pulled by another optical trap, as shown in Fig. 3g. During the stretching process, the oil droplets gradually became longer and thinner until it was split into two daughter droplets d_1_ and d_2_ (see ESI Video (Video 4)† for detailed splitting process of oil droplets). Moreover, further experiments indicate that the faster the stretching speed, the greater the difference in the size of the two daughter oil droplets (see Fig. S6 for the details in ESI†). Therefore, the size of the daughter oil droplets could be controlled by changing the stretching speed in the work. Additionally, it should be pointed out that, the appropriate volume ratio of emulsifier, toluene and water here was set as 1 : 5 : 50, which is very important to obtain splitting of oil droplets (see “Splitting of oil droplets” for discussions in ESI†).

### 3.3 Microreactors based on femtoliter/picoliter droplets

**Fig. 4:**
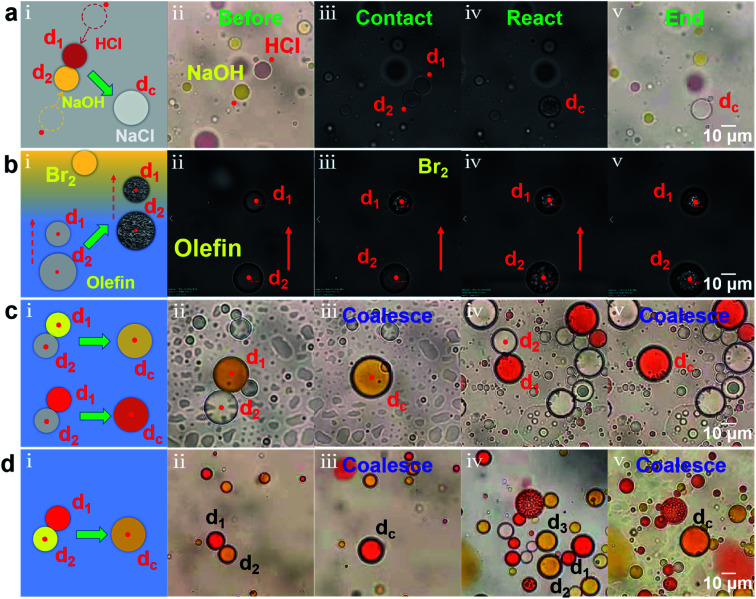
Microreactors based on femtoliter/picoliter droplets. (a) Acid–base neutralization reaction in water microdroplets dispersed in the silicone oil. (b) Olefin addition reaction in the oil microdroplets dispersed in the water. (c) Color mixing reactions through coalescing two oil droplets to dilute the dye concentration. (d) Color mixing reactions through coalescing two different dyes oil droplets. (i) Schematic diagram. (ii–v) Reaction process. In (c) and (d), the yellow droplet is Sudan I/dichloromethane, the red droplet is Sudan III/dichloromethane, and the colorless droplet is dichloromethane. The red dots represent the optical trap and the red arrow represents the transport direction.

The microdroplets have extreme mobility, elasticity, stability, universality and operability, which has large application prospects in chemical and biological process analysis, especially in reactions involving highly exothermic or toxic substances.^1,38^ We have realized the remotely controllable coalescence and splitting of microdroplets through the scanning optical tweezers system, so that the initiation and performance of the microreactions can be controlled on-demand and non-contact. Moreover, the reagent equivalent ratio can be strictly controlled by adjusting the reagent concentration and the size of the microdroplets for quantitative reaction analysis. Firstly, taking water droplets dispersed in the silicone oil as microreactors, an acid–base neutralization reaction was performed in Fig. 4a (see ESI Video (Video 5)† for detailed reaction process). The entire process of the reactions could be tracked and recorded in real time under the microscope with a water immersion objective with a magnification of 60 and a numerical aperture of 1.2 and a high-speed charge-coupled device camera. Fig. 4a(i) shows the schematic of the acid–base neutralization reaction. Using methyl red as an indicator, a red HCl water droplets d_1_ (1 mol L^−1^) and a yellow NaOH water droplets d_2_ (1 mol L^−1^) were approached, coalesced, and then reacted into a colorless neutral droplet d_*c*_ under the pushing of optical force. Fig. 4a(ii–v) shows the corresponding experimental process, including the process before reaction (ii, color image), in contact (iii), in reaction (iv) and after reaction (v, color image). From Fig. 4a and Video 5,† it can be also seen that at the moment of coalescence, the corresponding reaction began. Moreover, the reaction process was relatively rapid and violent, and obvious morphological changes could be observed (Fig. 4a(iv)). After the reaction, the shape of the water droplet recovered and the color faded (Fig. 4a(v)). Secondly, the olefin addition reaction was carried out in an oil droplet microreactor, as shown in Fig. 4b ((i) the schematic; (ii–v) the corresponding experimental images). Two optical traps are used to trap two octadecene oil droplets (d_1_, d_2_) and transport them to the upper bromine-containing area (Fig. 4b(ii)), where bromine can enter the oil droplets for addition reaction. The oil droplet d_1_ firstly reached the bromine-containing area and began to react (Fig. 4b(iii)). An obvious morphological change could be observed and the surface of the oil droplet d_1_ became irregular, which proves the occurrence of the reaction. When the droplet d_2_ also reached the bromine-containing area, the reaction happened and then its morphology became irregular (Fig. 4b(iv)). After the reaction, the morphological changes of two oil droplets were maintained (Fig. 4b(v)). Finally, color mixing reactions were performed in the oil droplets (Fig. 4c and d, also see ESI Video (Video 6)† for detailed color mixing reactions). On the one hand, the coalescence can be used to dilute the pigment concentration to reduce the color brightness, making the color lighter, as shown in Fig. 4c ((i) the schematic; (ii–v) the corresponding experimental images). When a yellow oil droplet d_1_ and a colorless oil droplet d_2_ were coalesced under the actions of two optical traps, the color of the formed droplet d_*c*_ changed from dark yellow (Fig. 4c(ii)) to light yellow (Fig. 4c(iii)). Similarly, after a red oil droplet d_1_ was coalesced with a colorless oil droplet d_2_, the color of the formed oil droplet d_*c*_ changed from orange-red (Fig. 4c(iv)) to light red (Fig. 4c(v)). On the other hand, two oil droplets of different colors could also be coalesced to produce a new color, as shown in Fig. 4d ((i) the schematic; (ii–v) the corresponding experimental images). When a red oil droplet d_1_ and a yellow oil droplet d_2_ were coalesced under the actions of two optical traps, an orange-red oil droplet d_*c*_ was produced (Fig. 4d(ii and iii)). One red oil droplet (d_1_) and two yellow oil droplets (d_2_, d_3_) were coalesced to produce an orange-yellow oil droplet d_*c*_ (Fig. 4d(iv and v)). Based on the above, various colors on demand can be obtained through the coalescence of microdroplets, providing new ideas for droplet imaging technology.^39^

## 4. Conclusion

In this article, with the assistance of a scanning optical tweezers system, two different schemes were proposed to stably trap and directionally transport oil droplets and water droplets with volumes ranging from femtoliters to picoliters in the liquid environment. On this basis, optically controlled coalescence and splitting of microdroplets were realized, and the corresponding conditions and realizing process were also explored. Studies have found that due to the directional arrangement of oil molecules on the surface, there was a strong electrostatic repulsive force *F*_e_ between two oil droplets, which could not be overcome by the optical force provided by optical tweezers and then hindered the coalescence of oil droplets. By increasing the ion concentration in the liquid environment or by exciting the fluorescence of oil droplets, oil droplets could successfully be coalesced under the pushing of optical forces. The former was owed to adsorbing ions with opposite charges into the surface of the oil droplets, and the latter was attributed to the weakening orderly arrangement of the oil molecules on the surface of oil droplets under the excitation of fluorophores. Both of them could reduce the electrostatic repulsive force *F*_e_ between the oil droplets. In contrast, due to the weak orderly arrangement of water molecules, the electrostatic repulsive force *F*_e_ between the water droplets was small enough to be ignored and thus the coalescence of water droplets could occur directly under the action of the optical forces. In addition, an oil droplet could be also stretched and split into two daughter oil droplets under the action of optical force by adding an emulsifier in the liquid environment. It is mainly because the emulsifier could be adsorbed on the surface of oil droplets to form an emulsified layer, which replaces the oil–water interface and reduces the interfacial tension of the oil droplet. Finally, the microdroplets were used as microreactors and their coalescence was applied into microreaction analysis. The acid–base neutralization reaction and the olefin addition reaction were completed in the water droplets and oil droplets, respectively. The color mixing reactions were also performed by coalescing a colorless oil droplet with another dye-containing oil droplet or by coalescing two different color oil droplets. The former could make the color of the dye lighter, and the latter could generate a new color, which realize a simple color controlling. Such optically controlled microreactors in femtoliter/picoliter droplets combine the miniaturization, flexibility, and functionality of the microdroplets, as well as the non-contact, non-invasiveness, flexibility and precise controllability of the optical manipulation, which will have huge application prospects in chemical and biological process analysis.

## Conflicts of interest

The authors declare no competing financial interest.

## Supplementary Material

RA-012-D2RA02230C-s001

RA-012-D2RA02230C-s002
