# Supplementary material for: Optically controlled coalescence and splitting of femtoliter/picoliter droplets for microreactors
Source: RSC Adv. 2022 Jun 22;12(29):18311–20. doi: 10.1039/d2ra02230c (PMC9215165; doi:10.1039/d2ra02230c)
Supplement: RA-012-D2RA02230C-s001 [file RA-012-D2RA02230C-s001.pdf]

Electronic Supplementary Material (ESI) for RSC Advances.
This journal is © The Royal Society of Chemistry 2022

# Supporting Information

# Optically controlled coalescence and splitting of femtoliter/picoliter droplets for microreactors

Mingcong Wen[1], Benjun Yao[1], Shun Yuan[1], Weina Zhang[2], Yao Zhang[3,*], Guowei Yang[1], and Hongxiang Lei[1,*]

[1]School of Materials Science and Engineering, Nanotechnology Research Center, State Key Laboratory of Optoelectronic Materials and Technologies, Sun Yat-sen University, Guangzhou 510275, China

[2]School of Information Engineering, Guangdong University of Technology, Guangdong Provincial Key Laboratory of Photonics Information Technology, Guangzhou 510006, China

[3]Institute of Nanophotonics, Jinan University, Guangzhou 511443, China

*Email address: zhyao5@jnu.edu.cn; leihx@mail.sysu.edu.cn.

## 1. Experimental setup

All the experiments in this paper were performed under the scanning optical tweezers system (Tweez250si, Europe) and the experimental setup is shown in Fig. S1. A 1064 nm laser beam (excited by a semiconductor-pumped Nd:$YVO_4$ laser) with the advantages of high penetration and low photothermal damage enters the acousto-optic deflector (AOD), which can modulate the temporal and spatial distribution of the laser beam in real time. Thus, several static or dynamic optical traps can be created and each of them can be further set with a trajectory by programming in Matlab. The modulated laser beam is expanded into a wide collimated laser beam by the beam expander. After reflected by a dichroic mirror, the beam is highly focused by the water immersion objective lens (60 ×, NA = 1.0) and formed one or several optical traps in the sample chamber for trapping and manipulating the microdroplets. The illuminating light is focused on the sample through a condenser, and real-time image and video are recorded by a high-speed charge-coupled device (CCD) camera. In addition, the system is equipped with a fluorescence excitation system. The light from a high-pressure mercury

lamp (HG-100W) is focused by a collector lens into the special fluorescent excitation block and a suitable excitation wavelength can be selected to excite the fluorescent microdroplets.

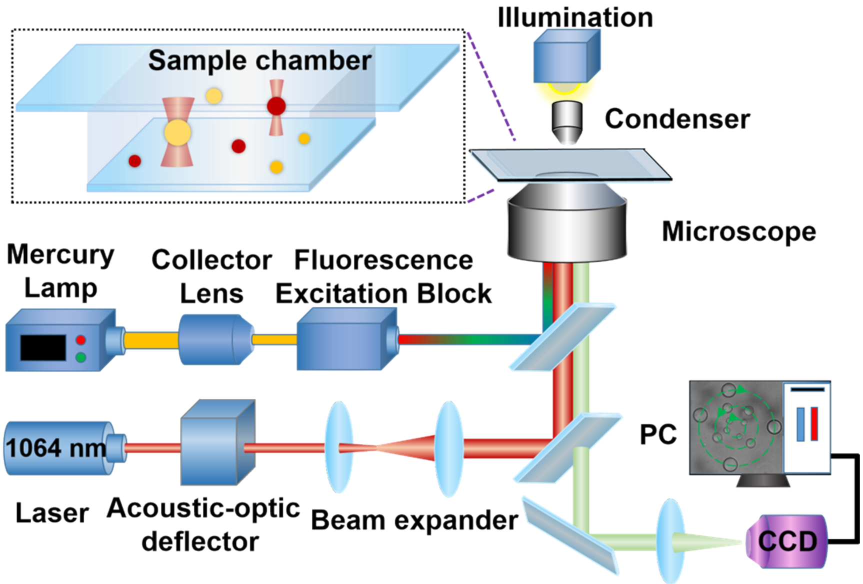


**Fig. S1. Schematic diagram of the experimental setup.** The enlarged picture on the upper left is the sample chamber.

**2. Analysis of the optical forces acted on microdroplets**

In optical tweezers system, a highly focused laser beam is typically used to trap and manipulate micro/nanoobjects through photon momentum transfer. The working principles are different for objects of different sizes and types. Generally, the applicable theoretical model can be selected according to the proportional relationship between the object radius $r$ and the laser wavelength $\lambda$. The laser wavelength used in the scanning optical tweezers system is 1064 nm, so ray optics (RO) model is used for objects with diameters ranging from several to tens of microns; the Rayleigh approximate electromagnetic (EM) model is used for objects with tens of nanometers in diameters. For the object with its diameter between the above two, the Lorentz-Mie scattering model can be used to investigate the optical force acted the object.

The size of the microdroplets studied and manipulated in this paper is at the micron level, so a ray optical model is suitable for analyzing the optical force acted on the microdroplet, as shown in Fig. S2. When the refractive index ($n_r$) of the droplet is larger than the refractive index ($n_i$) of the liquid environment, such as a silicone oil droplet in

water, the gradient force exerted on the droplet along the direction of the intensity gradient is larger than the scattering force along the light propagation direction, as shown in Fig. S2a. In this case, the droplets will move towards the center of the beam waist (i.e. the focus) and then trapped stably at the position. In short, the optical force exerted on the oil droplets in water is an attractive force. On the contrary, when $n_r$ is smaller than $n_i$, such as a water droplet in silicone oil, the optical force exerted on the droplet is a repulsive force (the scattering force is larger than the gradient force), as shown in Fig. S2b. In this case, the focused laser beam will push the water droplet away. Due to the different optical forces exerted on oil droplets and water droplets, different methods need to be adopted to achieve the trapping and manipulation of microdroplets.

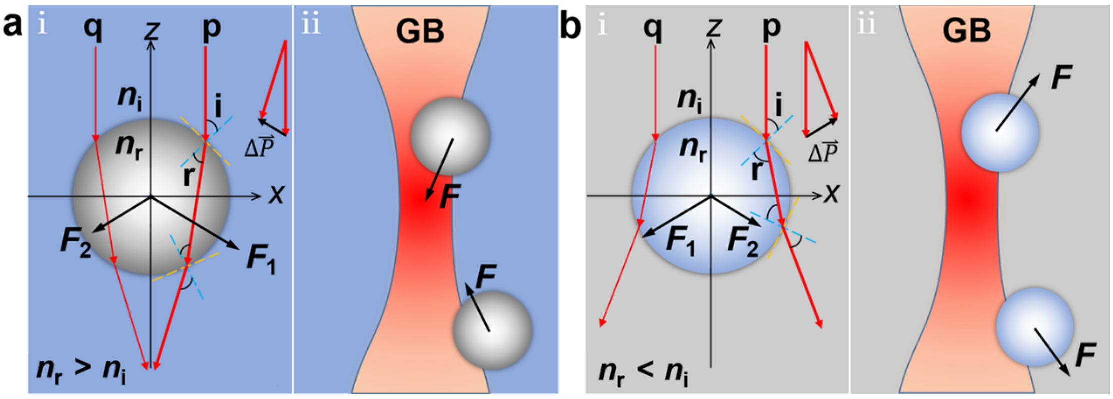


**Fig. S2. Analysis of the optical forces acted on microdroplets.** (a) Optical force acted on oil droplets in water medium. (b) Optical force acted on water droplets in oil medium. The red line represents the laser beam, its arrow refers to the propagation direction of light, the black arrow represents the force exerted on the droplet. The *i* and *r* are the incident angle and refraction angle, respectively, and $n_i$ and $n_r$ are the refractive index of the liquid environment and the droplet, respectively.

### 3. The limit of the background flow rate

At the constant viscosity of fluid environment in our experiment, the limit is mainly related to the laser power and the size of the water droplets. To demonstrate this conclusion, more experiments have been performed, as shown in Fig. S3. After a water microdroplet was trapped by a circular optical trap, a flow with a gradually increasing flow rate was introduced. Once the water droplet was about to escape the trap, the corresponding flow rate (averaged in multiple measurements) was taken as the limit of flow rate. In Fig. S3a, at the laser power of 80 mW, the water droplet with a diameter

of 25 μm was stably trapped within 0~6 s (i), and then started to escape from the optical trap at $t$ = 7 s and became free at $t$ = 13 s (ii). The limit of flow rate was calculated as about 4.2 μm/s, which is higher than the flow rate in Fig. 2f under the same condition. Reducing the laser power to 40 mW in Fig. S3b, the limit of flow rate was decreased to about 2.8 μm/s for the water microdroplet with the same size. This is mainly because the greater the laser power, the greater the optical force, the greater the viscous force and thus higher flow rate can be overcome. At the same laser power (80 mW), the limits of flow rate for the water droplets with diameters of 16 and 10 μm were measured as 5.5 (Fig. S3c) and 6.9 μm/s (Fig. S3d), respectively. It shows that at the same laser power, the limit of flow rate decreases with the increasing size of water microdroplet, which is in line with the Stock's law ($f = 6\pi r\eta v$, where $f$ is viscous force, $r$ is the water droplet radius, $\eta$ is the liquid viscosity, and $v$ is the flow rate).

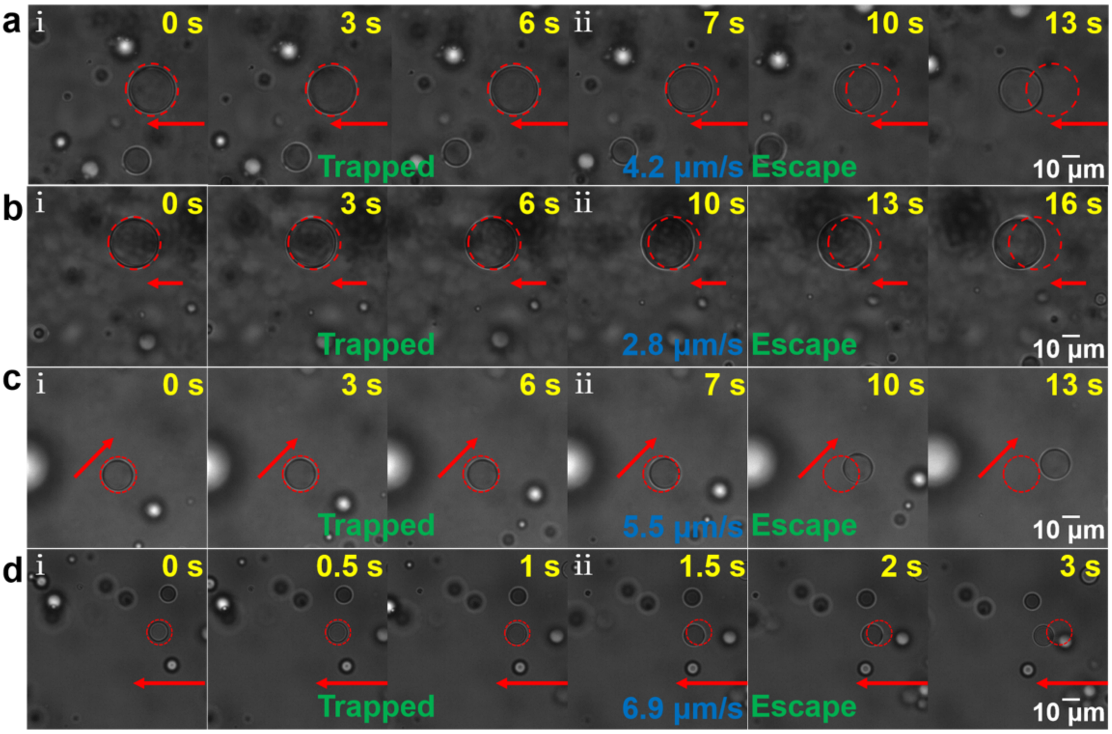


Fig. S3. Trapping of water droplets in the flow with a gradually increasing flow rate. The laser powers and the diameters of water microdroplets are 80 mW and 25 μm (a), 40 mW and 25 μm (b), 80 mW and 16 μm (c), 80 mW and 10 μm (d), respectively.

## 4. Manipulation of W/O/W and O/W/O microdroplets

It is also possible to control more complicated droplets, like W/O/W, O/W/O. To demonstrate this, more experiments have been performed, as shown in Fig. S4.

Fig. S4a(i) shows a schematic of W/O/W, which could be formed by dispersing oil microdroplets (silicone oil as an example) in water medium. When an optical trap (marked by a red dot) was set on the oil microdroplet part of the structure, the W/O/W could be transported directionally as a whole by the optical gradient force. Fig. S4a(ii) and S4a(iii) show the corresponding experimental results. For the case that the inner water microdroplet is much smaller than outer oil microdroplet, the optical trap did not exert an optical force on the inner water microdroplet because the trap was far from it. Thus, during the transporting process, the inner water microdroplet remained in the center of the outer oil microdroplet, as shown in Fig. S4a(ii). However, for the other case with an inner large water microdroplet, the inner water microdroplet was repulsed and moved away from the optical trap during the transporting process, as shown in Fig. S4a(iii). It was mainly because the optical trap acted an optical repulsive force on the water microdroplet when the trap was close to it.

Fig. S4b(i) shows a schematic of O/W/O, in which a smaller oil microdroplet is enclosed within a larger water microdroplet dispersed in oil medium. It could be formed by dispersing water microdroplets in oil medium (silicone oil as an example). An optical trap (red dot) can be set on the periphery of the water microdroplet or on the inner oil microdroplet. For the former, the O/W/O was transported directionally under an action of repulsive force from the optical trap, as shown in Fig. S4b(ii). In the case, the inner oil microdroplet could move to the edge of the water microdroplet due to the attractive force from the optical trap. For the latter, the inner oil microdroplet could be transported controllably within the outer water microdroplet by the attractive force from the optical trap, such as the counterclockwise rotation shown in Fig. S4b(iii).

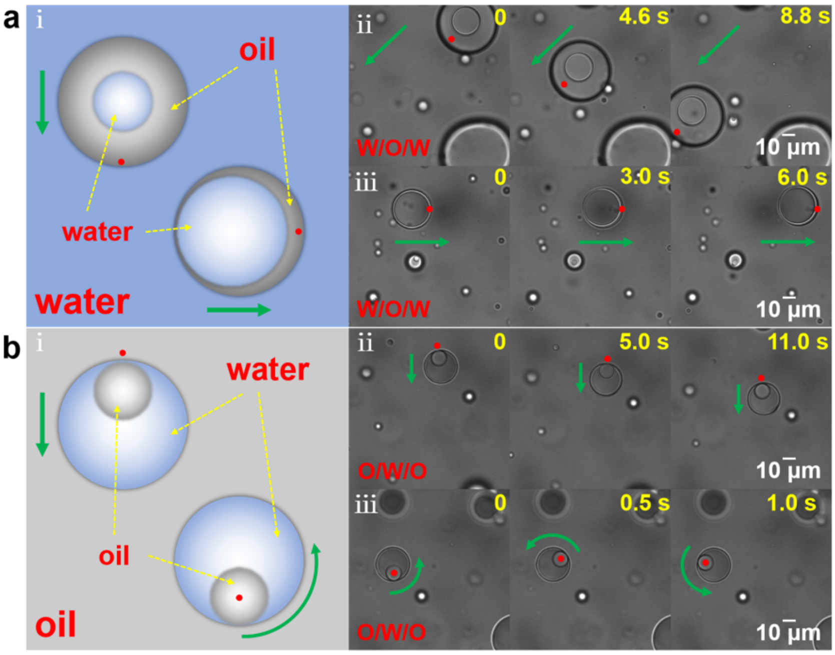


Fig. S4. Manipulation of more complicated microdroplets with W/O/W (a) and O/W/O (b). (i) Schematic diagram. (ii,iii) Transportation process. The red dot represents an optical trap and the green arrow represents the transportation direction.

**5. Coalescence of oil droplets in ionic solution**

Due to the directional arrangement of the hydrophilic and hydrophobic groups of the surface oil molecule, the positive and negative charge centers on the surface of the oil droplets deviate, which makes the surface of oil droplets carry a certain charge. Thus, an electrostatic repulsive force $F_e$ is generated between the oil droplets approaching each other. The optical force exerted on the droplets by the optical tweezers is about piconewton order, which can provide a dynamic force to control the motion of the droplets but cannot overcome the force $F_e$. Therefore, the coalescence of oil droplets will not occur under the pushing of optical force unless the force $F_e$ is overcome. Fig. S5a shows the approaching, contacting, superimposing and separating process of two oil droplets dispersed in deionized water under the actions of optical forces. In this case, even if the laser power was adjusted to the maximum, it is still insufficient to overcome the electrostatic repulsive force. Thus, two contacted oil droplets could not be coalesced but superimposed. This superposition could be separated at any time. The coalescence of oil droplets can be achieved by absorbing the ions on the surface. When the oil droplets are dispersed in the ionic solution, some oppositely charged ions will be adsorbed on the surface of oil droplets to reduce the electrostatic repulsive force $F_e$

between the droplets, which will facilitate the occurrence of coalescence. To demonstrate this conclusion, more experiments have been performed. As an example, Fig. S5b–i shows the coalescence of silicone oil droplets in different ionic solutions. When the ion concentration reached a certain value, the electrostatic repulsive force $F_e$ between the oil droplets was reduced to a value that could be overcome by optical forces and thus the oil droplets could be coalesced under the pushing of the optical forces. Otherwise, the coalescence did not occur. Specifically, when the concentrations of HCl, NaCl, KCl, $CaCl_2$, NaOH, $H_2SO_4$, $Na_2SO_4$ and $K_2SO_4$ solutions were greater than 0.0025, 0.1, 0.25, 0.005, 0.1, 0.001, 0.05 and 0.1 mol/L, respectively, the silicone oil droplets dispersed in the ionic solution can be coalesced under the pushing of optical force (Fig. S5b(i)–i(i)); when the concentration of HCL, NaCl, KCl, $CaCl_2$, NaOH, $H_2SO_4$, $Na_2SO_4$ and $K_2SO_4$ solutions were less than 0.001, 0.075, 0.1, 0.0025, 0.075, 0.0005, 0.03 and 0.05 mol/L, respectively, the droplets could not be coalesced after contacting (Fig. S5b(ii)–i(ii)). From the above, it can be concluded as following. Firstly, from the coalescence results of oil droplets in HCl, NaCl, KCl $H_2SO_4$, $Na_2SO_4$ and $K_2SO_4$ solutions, the cation in the solutions plays a key role in reducing the electrostatic repulsive force $F_e$ between oil droplets. The coalescence of oil droplets in NaCl and NaOH solution is similar, which can also prove the above conclusion. Secondly, for the same concentration, the smaller the ion radius, the stronger the effect of reducing the force $F_e$ between oil droplets. This is mainly because with the same concentration, the smaller the radius, the closer the cation is to the surface of the oil droplet and the more $F_e$ is offset, which can be concluded from the coalescence results of oil droplets in HCl, NaCl, KCl, $H_2SO_4$, $Na_2SO_4$ and $K_2SO_4$ solutions. Thirdly, for the same concentration and ion radius, the more the charges the ions carry, the more $F_e$ is offset. The cation concentration required for the coalescence of silicone oil droplets in $CaCl_2$ solution is much smaller than that in KCl solution, which can prove the above conclusion.

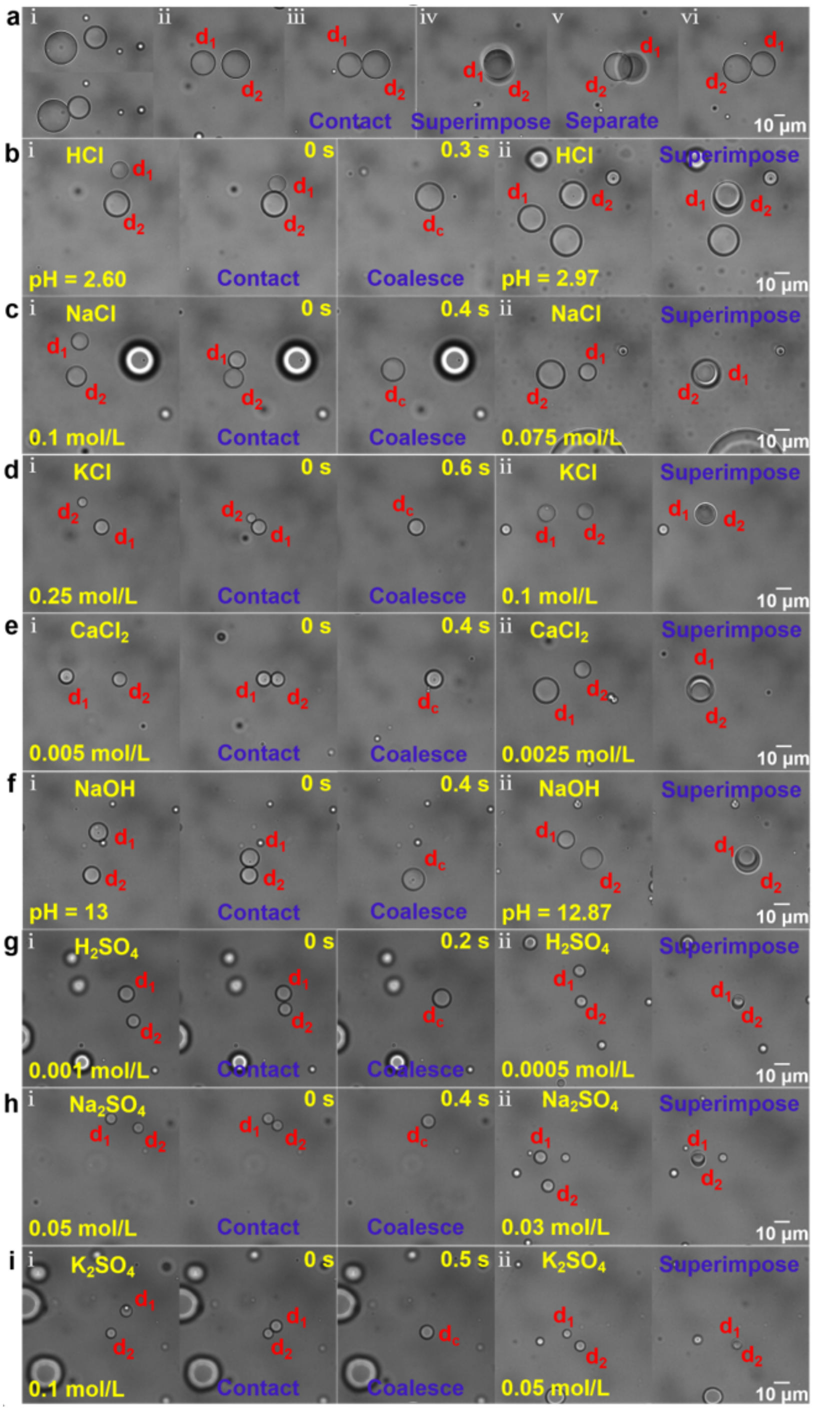


**Fig. S5. Coalescence of silicone oil droplets in different ionic solutions.** (a) Deionized water. (b) HCl solution. (c) NaCl solution. (d) KCl solution. (e) $CaCl_2$ solution. (f) NaOH solution. (g) $H_2SO_4$ solution. (h) $Na_2SO_4$ solution. (i) $K_2SO_4$ solution.

## 6. Splitting of oil droplets

Fig. S6 shows the splitting of toluene oil droplets dispersed in water containing OP-10 emulsifier under the pulling of optical force. One end of the toluene oil droplets was fixed by one optical trap, and the other end was pulled by another optical trap. The oil

droplets gradually became flat and then began to become thinner in its middle under the pulling of optical force. When it was stretched to a critical state, the oil droplet was split from the middle into two daughter oil droplets. Then the two daughter oil droplets were aggregated into spherical shapes under the action of surface tension and optical attractive force. The splitting results with different stretching speed in Fig. S6a-c indicate that the faster the stretching speed, the greater the difference in the sizes of two daughter oil droplets. It is mainly because, when the stretching speed is too fast, the oil droplet had been split into two daughter oil droplets before the diffusion was completed. If the stretching speed is too slow, the surrounding free oil droplets might be coalesced with the trapped droplet in the stretching process, making the total volume of the two daughter oil droplets larger than that of the parent oil droplet, as shown in Fig. S6c.

Additionally, it should be pointed out that, an appropriate amount of emulsifier is very important to obtain splitting of oil droplets. Here, volume ratio of emulsifier, toluene and water chosen here was set as 1:5:50. When a smaller amount of emulsifier was used (such as the volume ratio of 1:6:60), the optical force acted on the oil droplets was not enough to overcome their interfacial tension and thus the oil droplets could not be split. When a larger amount of emulsifier was used (such as the volume ratio of 1:3:30), excessive emulsifier would gather around the oil droplets to form a “tight” structure. On the one hand, this “tight” structure could reduce the light transmittance of the sample and make the visual field darker, which is not conducive to experimental observation and image acquisition. On the other hand, the “tight” accumulation of emulsifiers increased the viscous force acted on oil droplets during tensile deformation, which hindered the splitting of oil droplets.

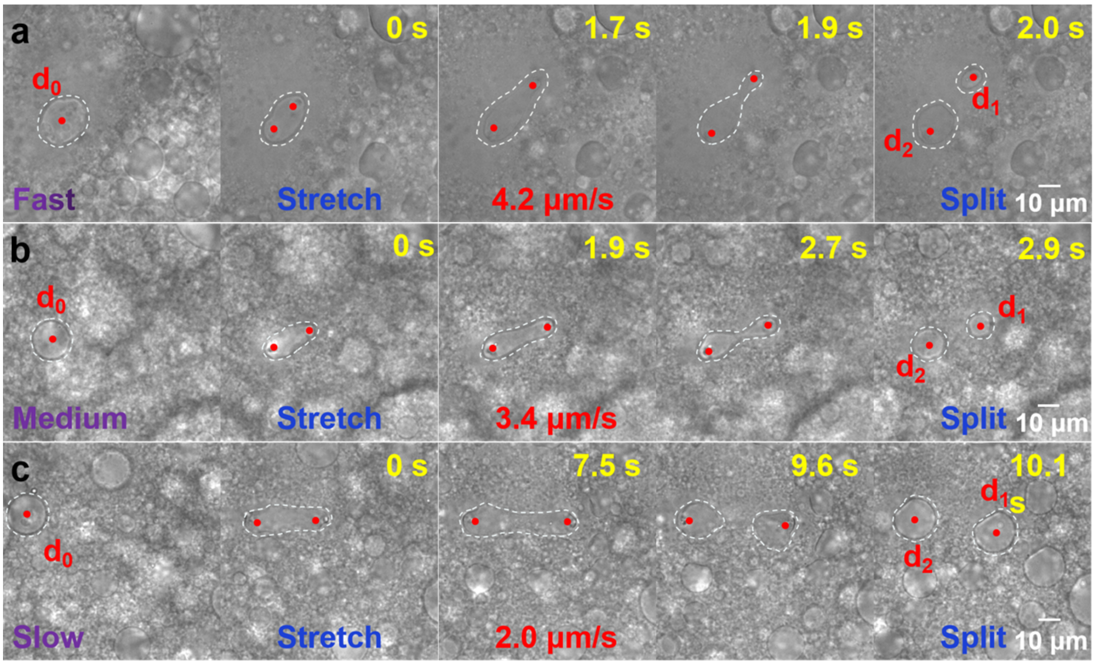


**Fig. S6. Splitting of oil droplets at different stretching speed.** (a) 4.2 μm/s. (b) 3.4 μm/s. (c) 2.0 μm/s.

**Supplementary References:**

[1] K. Dholakia, P. Zemanek, Gripped by light: optical binding. *Rev. Mod. Phys.* **2010**, 82, 1767-1791.

[2] O. M. Marago, P. H. Jones, P. G. Gucciardi, G. Volpe, A. C. Ferrari, Optical trapping and manipulation of nanostructures. *Nat Nanotechnol.* **2013**, 8, 807-819.

[3] Y. J. Yang, Y. X. Ren, M. Z. Chen, Y. Arita, C. Rosales-Guzman, Optical trapping with structured light: a review. *Adv. Photon.* **2021**, 3, 034001.

[4] M. Daly, M. Sergides, S. N. Chormaic, Optical trapping and manipulation of micrometer and submicrometer particles. *Laser Photonics Rev.* **2015**, 9, 309-329.
